# Differentiation of *Bulinus* *senegalensis* and *Bulinus* *forskalii* Snails in West Africa Using Morphometric Analysis

**DOI:** 10.1007/s11686-024-00830-1

**Published:** 2024-03-19

**Authors:** Peter S. Andrus, Ebrima Joof, Christopher M. Wade

**Affiliations:** School of Life Sciences, University of Nottingham, NG7 2RD Nottingham, UK

**Keywords:** *Bulinus* snails, Schistosomiasis, Morphometrics, Shell morphology

## Abstract

**Purpose:**

Accurate identification of medically important intermediate host and vector species is crucial for understanding disease transmission and control. Identifying *Bulinus* snails which act as intermediate host species for the transmission of schistosomiasis is typically undertaken using conchological and genital morphology as well as molecular methods.

**Methods:**

Here, a landmark-based morphometric analysis of shell morphology was undertaken to determine its utility to distinguish the closely related and morphologically similar sister species *Bulinus senegalensis* and *Bulinus forskalii*. The method was developed to increase the accuracy of conchological morphology methods to identify *Bulinus* species in the field. Both species are found in West Africa, but only *B. senegalensis* is implicated in the transmission of urogenital schistosomiasis.

**Results:**

We found when scaled down to the same length, 3-whorl and 4-whorl (juvenile) *B. senegalensis* shells had a longer spire, narrower body whorl and shorter aperture than *B. forskalii*. In contrast, 5-whorl (adult) *B. senegalensis* had a shorter spire, but still had a shorter aperture and narrower body whorl than *B. forskalii*. Canonical Variate Analysis (CVA) showed minimal overlap between *B. senegalensis* and *B. forskalii* for 3-whorl and 4-whorl shells, with a clear separation for 5-whorl shells. Overall, *B. senegalensis* had a consistently shorter aperture size and narrower body whorl than *B. forskalii* for all development stages. Spire length was variable depending on the stage of development, with 3-whorl and 4-whorl shells having the opposite trends of adult shells.

**Conclusions:**

Our study demonstrates the applicability of landmark-based morphometrics in distinguishing the medically important, *Bulinus senegalensis* from its morphologically similar sister species, *Bulinus forskalii*. We recommend using measurements based on spire length, penultimate whorl length, body whorl width and aperture size to differentiate *B. senegalensis* and *B. forskalii*, when used with the appropriate information for each shell’s development stage.

**Supplementary Information:**

The online version contains supplementary material available at 10.1007/s11686-024-00830-1.

## Introduction

*Bulinus* (Gastropoda: Bulinidae) [1, 2] is a genus of tropical freshwater snails with a sinistral shell, found throughout Africa and in neighbouring tropical islands, South West Asia, Arabia and the Mediterranean region [3]. The genus is of significant medical (and veterinary) importance as its species act as intermediate hosts for the causative agents of urogenital schistosomiasis (*Schistosoma haematobium*) and intestinal schistosomiasis (*S. guineensis* and *S. intercalatum*) in humans and in ruminants (*S. bovis*) as well as other trematode species of veterinary importance (e.g., *Paramphistomum* spp.) [4–8]. Several *Bulinus* species are endemic to West Africa including *B. jousseaumei, B. globosus*, *B. umbilicatus* (*B. africanus* group; short-spire, large body whorl), *B. forskalii*, *B. senegalensis* (*B. forskalii* group; long-spire, small body whorl) and *B. truncatus* (*B. truncatus*/*tropicus* complex; short-spire, large body whorl). Of these, *B. globosus*, *B. truncatus* and *B. senegalensis* act as intermediate hosts for *S. haematobium* in Africa [9]. *Bulinus forskalii* was reported as a potential host of *S. haematobium* in the Niger Valley by Labbo et al. (2007) [10], but only five out of 21,820 snails were found naturally infected and there is uncertainty about the means of identification and the role *B. forskalii* has (if any) in the transmission of *S. haematobium* [11]. *Bulinus forskalii* is however an intermediate host for the human parasites *Schistosoma intercalatum* and *S. guineensis* [5–7]. They are less prevalent than *S. haematobium* and only occur in specific areas of the Lower Guinea region and Democratic Republic of the Congo [5, 7].

**Fig. 1:**
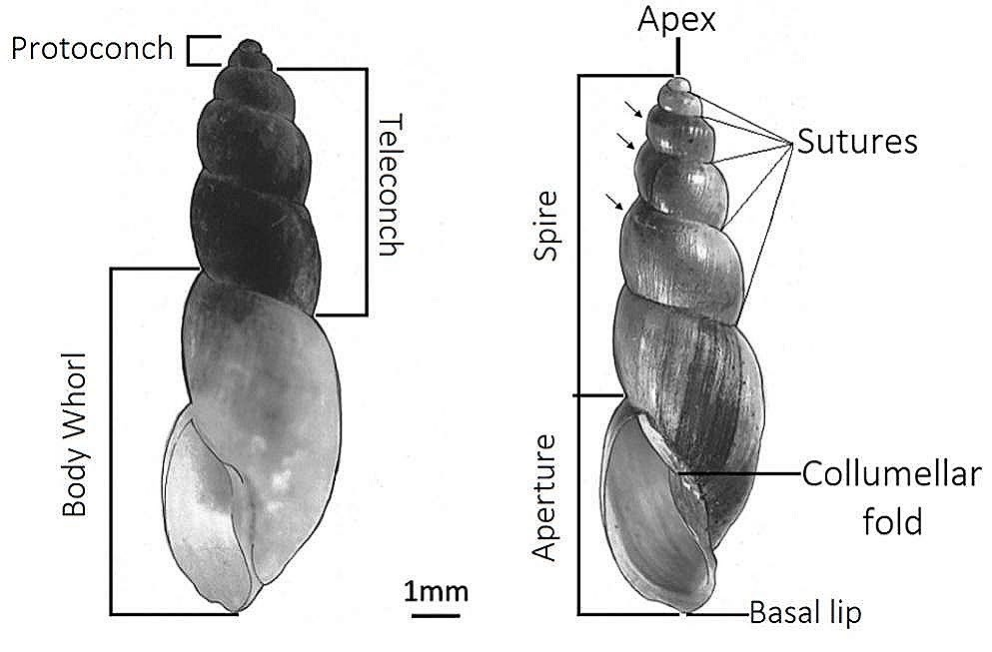
Shell morphologies of adult *Bulinus senegalensis* (left) and *B. forskalii* (right). Arrows indicate the shoulder angles found only in adult *B. forskalii* shells

In West Africa, *B. senegalensis* and *B. forskalii* often co-inhabit the same permanent (irrigation canals, rice fields and swamps) and seasonal (laterite pools) habitats [12]. Given the shell morphological similarity of these two species (Fig. 1), it is often exceedingly difficult to determine which species of *Bulinus* is present in a locality and therefore what risk a *Bulinus* population may pose to a local community. The ability to identify medically and veterinary relevant species is important. Bulinid snail species are commonly categorised and identified using a combination of genital, radula and shell morphology [13], with taxonomic guides traditionally using morphological characters to identify species [1, 14–19]. Genital morphology is the most dependable as complementary copulatory organs are essential for intraspecies mating [20]. However, identifying snails using genital morphology requires time and expertise, as the genitals need to be extracted from the snail, prepared, and mounted under a microscope to be observed and measured. Molecular identification methods (e.g., COI barcoding) provide a reliable alternative to traditional morphological identification methods but are time consuming, expensive and require training and facilities. A good identification method is one with high reliability (like genital morphology and COI barcoding), but with less requirement for training, equipment, and time.

Shell morphological identification by itself does not meet all of these criteria as shell morphology is a variable characteristic in snails and can be affected by environmental factors such as water flowrate, temperature, predation and parasitism [21–28]. As a result, taxonomists using only shell morphology typically overestimate the number of species within a genus due to the variety of shell morphotypes exhibited in one species, with these taxonomical mistakes only being clarified using more advanced techniques such as genital morphology or COI barcoding. However, the simplicity of shell morphological identification, its direct applicability and low cost make it a desirable option for the identification of snails. For example, a simplistic morphological characteristic for the differentiation of *B. senegalensis and B. forskalii* shells is the presence of a shoulder angle on the early whorls of *B. forskalii* shells; shoulder angles are absent in *B. senegalensis* [3, 19]. However, due to shell plasticity, the shoulder angles are not always present in *B. forskalii* shells or can be obscured by detritus. Moreover, as *Bulinus* snails develop from juveniles to adults their shells grow, the number of whorls increase from three to five and the shoulders angles become more prominent [29]. Therefore, the stages of development (or number of whorls) can affect how similar *B. senegalensis and B. forskalii* look, with three whorl juveniles having underdeveloped characteristics and being harder to distinguish from each other. One way to address the issues of using morphological characteristics for *Bulinus* identification is to employ geometric, landmark-based morphometric techniques [30, 31]. The addition of geometric, landmark-based morphometrics could improve how reliably and accurately one can distinguish snail species that share similar shell morphologies.

Here we investigate the use of landmark based morphometric methods for the differentiation of *Bulinus senegalensis and B. forskalii* using *B. truncatus* as a comparative outgroup. Specimens were examined from multiple locations across The Gambia, a country endemic with both *B. senegalensis* and *B. forskalii*, with additional museum specimens also examined to add variation from other African countries.

## Materials and Methods

### Sample Collection, Preparation, and Photography

*Bulinus senegalensis*, *B. forskalii* and *B. truncatus* specimens were collected from permanent and seasonal sites across The Gambia over the course of two years (2017–2019) [32]. In total, 15 sites, each with only a single *Bulinus* species present, were selected for this study (Table 1). Additional *B. senegalensis* and *B. forskalii* samples were provided by the London Natural History Museum (NHM), with dry shell collections provided by Jonathan Ablett and wet collections provided by Dr Aidan Emery. These NHM *Bulinus* collections were from multiple African countries, with *B. senegalensis* available from three countries and *B. forskalii* available from 16 countries (Table 2). *Bulinus* samples from The Gambia (Table 1) were identified using cytochrome oxidase I (COI) DNA barcoding [32]. *Bulinus* samples from the NHM (Table 2) were identified using both genital and shell morphology upon collection by their respective collectors (D. S. Brown; G. Mandahl-Barth; O. F. Müller; C. A. Wright & S. R. Smithers). The Gambian samples were stored in absolute ethanol, while the NHM samples were kept either as dry collections or preserved in IMS (wet collection). The NHM samples were not suitable for molecular analysis since only shells were available for specimens in the dry collection and specimens in the wet collection were preserved in IMS and not amplifiable in PCR.

Samples with no (or minimal) damage to their shells were selected for photography. This limited the number of viable shells collected from The Gambia due to their fragility as a result of ethanol preservation. Photographs were taken using a dissection microscope with a 64MegaPixel mobile phone camera attached. All shells were positioned and photographed the same way, with a 1 mm, 5 and 10 mm scalebar present; shell length (L1-L3) and shell width (L7-L8) were measured digitally using the software Digimizer v5.7.2 [33].

**Table 1:** List of collection sites from The Gambia (Ecology: A = seasonal laterite pool; B = stream; C = irrigated rice field). The *Bulinus senegalensis* (*n* = 90), *Bulinus forskalii* (*n* = 55) and *Bulinus truncatus* samples listed were taken from Joof et al. (2021)

	Species	Location	Ecology	Specimens (n)	Number of Whorls		
					3	4	5
1	*B. senegalensis*	Bajakunda	A	15	10	5	0
2	*B. senegalensis*	Changai 2	A	10	0	5	5
3	*B. senegalensis*	Diabugu Basilla	A	10	5	5	0
4	*B. senegalensis*	Kuwonku	A	10	5	5	0
5	*B. senegalensis*	Madina Nfally 2	A	10	5	5	0
6	*B. senegalensis*	Sare Jabel	A	10	10	0	0
7	*B. senegalensis*	Sare Madi Ganteh	A	10	5	5	0
8	*B. senegalensis*	Sutukonding	A	15	0	10	5
1	*B. forskalii*	Bansang	C	10	10	0	0
2	*B. forskalii*	Basse Kabakama	B	5	5	0	0
3	*B. forskalii*	Choya	B	10	5	5	0
4	*B. forskalii*	Dalaba	B	5	5	0	0
5	*B. forskalii*	Kuntaur	C	5	5	0	0
6	*B. forskalii*	Misra Ba Mariama	B	10	10	0	0
7	*B. forskalii*	Sotuma Sire	B	10	5	5	0
1	*B. truncatus*	Pacharr	C	5	5	0	0

**Table 2:** List of *Bulinus* samples photographed from the London Natural History Museum collections. *Bulinus senegalensis* (*n* = 30) and *Bulinus forskalii* (*n* = 55)

	Species	Country	Collection (Wet or Dry)	Specimens	Number of Whorls			
					3	4	5	
1	*B. senegalensis*	The Gambia	(Dry)	5	3	1		1
2	*B. senegalensis*	The Gambia	(Wet)	18	14	4		0
3	*B. senegalensis*	Nigeria	(Wet)	4	3	0		1
4	*B. senegalensis*	Senegal	(Wet)	3	0	2		1
1	*B. forskalii*	Angola	(Dry)	6	0	3		3
2	*B. forskalii*	Democratic Republic of Congo	(Dry)	3	0	0		3
3	*B. forskalii*	Egypt	(Dry)	3	0	0		3
4	*B. forskalii*	Ethiopia	(Wet)	4	2	2		0
5	*B. forskalii*	The Gambia	(Wet)	9	6	3		0
6	*B. forskalii*	Kenya	(Dry)	2	2	0		0
7	*B. forskalii*	Mauritius	(Dry)	2	2	0		0
8	*B. forskalii*	Rwanda	(Dry)	1	1	0		0
9	*B. forskalii*	S. Africa	(Dry)	3	0	3		0
10	*B. forskalii*	S. Sudan	(Dry)	1	1	0		0
11	*B. forskalii*	Senegal	(Dry)	6	1	2		3
12	*B. forskalii*	Seychelles	(Dry)	4	1	0		3
13	*B. forskalii*	Sierra Leone	(Dry)	2	2	0		0
14	*B. forskalii*	Somalia	(Dry)	5	1	4		0
15	*B. forskalii*	Tanzania	(Dry)	1	0	0		1
16	*B. forskalii*	Uganda	(Dry)	3	0	2		1

### Morphometric and Data Analysis

**Fig. 2:**
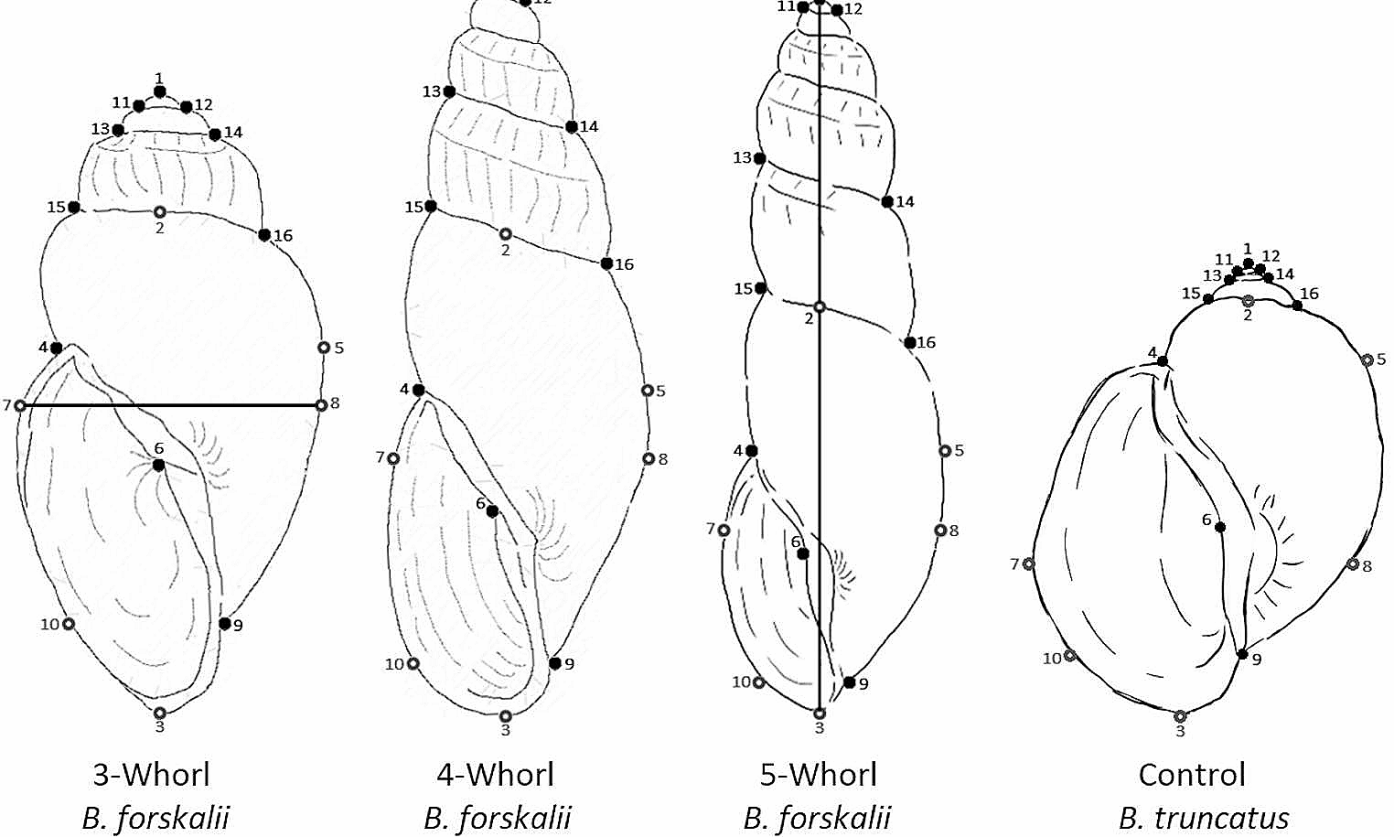
Example of the fixed landmarks (black) and semi-landmarks (grey) placed on *Bulinus forskalii* and *B. truncatus* shells (not to scale). Additionally, adult *Bulinus truncatus* shells were also included as a comparative outgroup. L1-L3 were used to measure shell length (or height) and L7-L8 were used to measure shell width. Landmark placements, L1: tip of the Apex; L2: middle of the Body whorl and Teleconch suture (aligned with L1); L3: bottom of Basal lip (aligned vertically with L1 and L2); L4: meeting point between the Aperture and Body whorl; L5: aligned horizontally on the right of L4; L6: placed in the Columellar fold; L7-L8: maximum horizontal width of the shell; L9: meeting point between the Columella and Body whorl; L10: aligned horizontally on the left of L9; L11: left suture between the Apex and Protoconch; L12: right suture between the Apex and Protoconch; L13: left suture between the Protoconch and Teleconch; L14: right suture between the Protoconch and Teleconch; L15: left suture between the Teleconch and Body whorl; L16: right suture between the Teleconch and Body whorl

To minimise errors associated with shell plasticity, we took several precautionary measures in our morphometric analysis: (i) using a comparative outgroup; (ii) incorporating multiple individuals from diverse populations to average out the plastic variation within the dataset; (iii) separating adult and juveniles specimens by whorl number to reduce the morphological disparities between shells at different stages of development; (iv) using a Procrustes’ fit analysis to eliminate undesirable effects of translation, rotation, and scaling from our landmark placement; and (v) utilising outlier detection to exclude individuals with significant morphological deviations from the final analysis. Shell photographs were imported into the tpsDig2 v2.31 program [34] and each image was digitised using 16 landmarks comprised of ten fixed landmarks (anatomically meaningful) and six semi-landmarks (non-anatomically meaningful) to collect two-dimensional coordinate data (Fig. 2). The placement of our 16 landmarks was inspired by the *Bulinus* shell measurements used by Stothard et al. (1997) [17] and the landmarks used by Hammoud et al. (2022) [27]. This coordinate data was stored in a TPS file, and each sample was scaled and had a unique specimen ID, which displayed species (Bsen, Bfor or Btru), number of whorls (3W, 4W or 5W) and location (e.g., ID = Bsen-3W-Changai-1). The TPS file was then imported into the MorphoJ v1.07 program [35]. The data was treated as symmetrical due to the bilateral symmetry for some of the landmarks (e.g., LM4&5, LM7&8, LM9&10, LM11&12 and LM13&14; Fig. 2). Each specimen was grouped by species (*B. senegalensis, B. forskalii* or *B. truncatus*), with the groups being defined by genetic (Gambian dataset) or genital morphology (NHM dataset) identification methods. The specimens were then partitioned based on the number of whorls they had (3-whorl, 4-whorl and 5-whorl). Each of the whorl datasets had a Procrustes’ fit analysis performed and shell shape was assessed using a Canonical Variate Analysis (CVA) across all landmarks using 10,000 permutations.

A Canonical Variate Analysis (also known as Canonical Correlation Analysis or Linear Discrimination Analysis) is a statistical method used to examine the relationship between two sets of variables. In our scenario, the landmark coordinate data is the independent variable and the species groups are the dependent variables. Our CVA is multivariate analysis that extracts crucial information (named canonical variables) from complicated datasets and is able to uncover patterns not immediately apparent in the raw data. These canonical variables are linear combinations of the original variables and are selected based on their efficacy in explaining the variation between the two datasets. The first canonical variable (CV1) explains the most variation, while the following canonical variable (CV2) explains the second most. A canonical variate analysis was preferred over alternative multivariate statistical methods, such as a Principal Component Analysis (PCA), due to its optimisation for classifying and discriminating groups within large datasets.

In addition to the morphometric analysis, the mean distances between informative landmarks were taken for both *B. senegalensis* and *B. forskalii* shells using Digimizer v5.7.2 [33]. However, on average the *B. senegalensis* shells were larger than the *B. forskalii* shells. Therefore, the shell length for both species was first scaled down to a standardised length depending on development stage (3-whorl = 2 mm; 4-whorl = 4 mm; 5-whorl = 6 mm). The informative landmarks selected were informed by the CVA analysis (Supplementary Fig. 1). A Mann–Whitney U test was chosen as the distance data was interval and had a non-normal distribution. The Mann–Whitney U test was performed in SPSS v26 (IBM, Armonk, USA) [36] and was used to see whether there was a significant difference in the landmark distances between the two species.

## Results

**Fig. 3:**
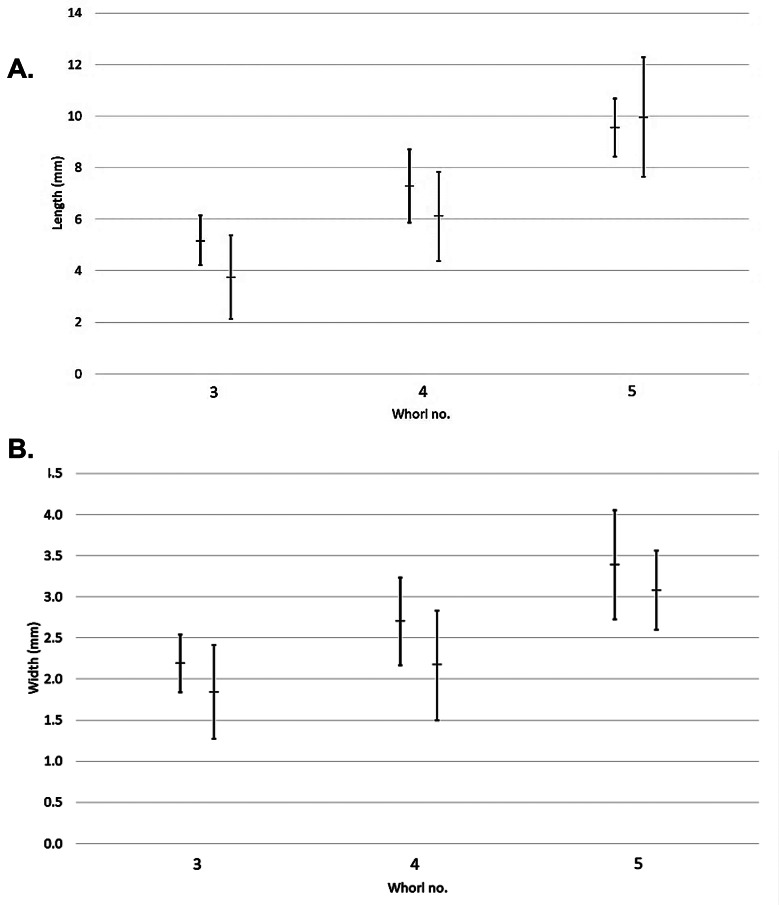
(**A**) Mean shell lengths (L1-L3) and (**B**) mean shell width (L7-L8) (error bars: standard deviation) of all *B. senegalensis* (*n* = 120) (left) and *B. forskalii* (*n* = 110) (right)

The Gambian sites had a mixture of different developmental stages of *Bulinus* (3-whorl, 4-whorl, 5-whorl), with some sites only having one developmental stage and others having all developmental stages (Table 1). More juvenile snails (3-whorl or 4-whorl) were present than adult snails (5-whorl). The mean length (measurement L1-L3) of a 3-whorl shell was 5.2 mm (± 1) for *B. senegalensis* (*n* = 60) and 3.8 (± 1.6) for *B. forskalii* (*n* = 64; Fig. 3a). The mean width (measurement L7-L8) of a 3-whorl shell was 2.2 mm (± 0.4) for *B. senegalensis* and 1.8 mm (± 0.6) for *B. forskalii* (Fig. 3b). For 4-whorl shells, the mean length was 7.3 mm (± 1.2) for *B. senegalensis* (*n* = 47) and 5.9 mm (± 1) *for B. forskalii* (*n* = 29; Fig. 3a). The mean width of a 4-whorl shell was 2.7 mm (± 0.5) for *B. senegalensis* and 2.2 mm (± 0.6) for *B. forskalii* (Fig. 3b). Finally, for 5-whorl shells the mean length was 9.7 mm (± 0.9) for *B. senegalensis* (*n* = 13) and 10 mm (± 1.1) for *B. forskalii* (*n* = 17; Fig. 3a). The mean width of a 5-whorl shell was 3.1 mm (± 0.4) for *B. senegalensis* and 3.4 mm (± 0.5) for *B. forskalii* (Fig. 3b).

There were no noticeable differences between the mean length and widths of *B. senegalensis and B. forskalii* shells (Fig. 3a/3b), though the Gambian samples showed on average that *B. forskalii* was shorter than *B. senegalensis* (Supplementary Tables 1 and 2).

**Fig. 4:**
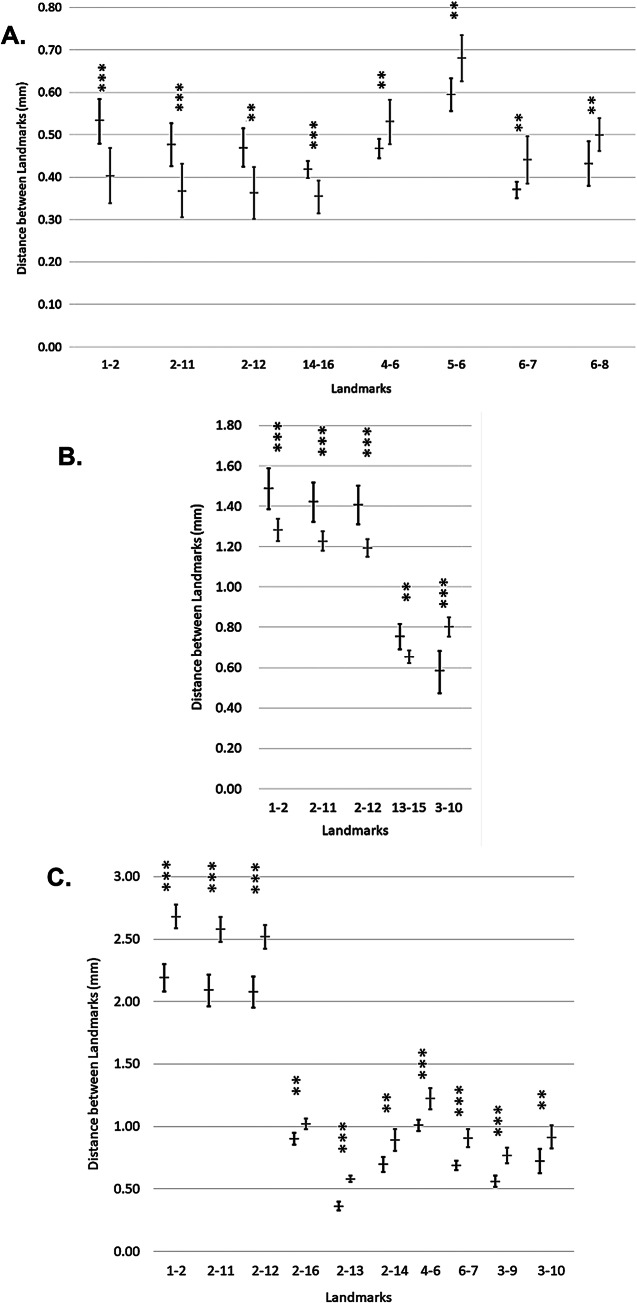
Mean distances between landmarks (error bars: standard deviation) of (**A**) 3-whorl juvenile, (**B**) 4-whorl and (**C**) 5-whorl, adult *B. senegalensis* (left) and *B. forskalii* (right) shells scaled down to the same length (3W = 2 mm; 4W = 4 mm; 5W = 6 mm). Only significantly different distances between informative landmarks are shown. A Mann-Whitney U test was used (* <0.05; ** <0.01; *** <0.001) and performed in SPSS v26

When the samples were scaled down to the same shell length and the distance between specific landmarks were measured, we found clear differences in shell morphology. The 3-whorl shells had eight significant differences between landmark measurements. *Bulinus senegalensis* had a longer distance from L1 to L2, L2 to L11 and L2 to L12 of the spire; a longer distance from L14 to L16 of the penultimate whorl; a narrower distance from L5 to L6 and L6 to L8 of the body whorl; a shorter distance from L4 to L6 and L6 to L7 of the aperture than *B. forskalii* (Fig. 4a). These were the only significant measurements found that can distinguish 3-whorl *B. senegalensis* from *B. forskalii*. The 4-whorl shells had five significant differences between landmark measurements; *B. senegalensis* had a longer distance from L1 to L2, L2 to L11 and L2 to L12 of the spire; a longer distance between L13 to L15 of the penultimate whorl and a shorter distance from L3 to L10 of the aperture than *B. forskalii* (Fig. 4b). Finally, the 5-whorl shells had ten significant differences between landmark measurements, though some of the trends were the opposite to 3-whorl and 4-whorl shells. Adult, 5-whorl *B. senegalensis* had a shorter distance from L1–L2, L2–L11 and L2–L12 of the spire; a shorter distance from L2 to L13 and L2 to L14 of the penultimate whorl than *B. forskalii*. However, *B. senegalensis* still had a narrower distance from L2 to L16 of the body whorl and a shorter distance from L3 to L9, L3 to L10, L4 to L6 and L6 to L7 of the aperture than *B. forskalii* (Fig. 4c).

**Fig. 5:**
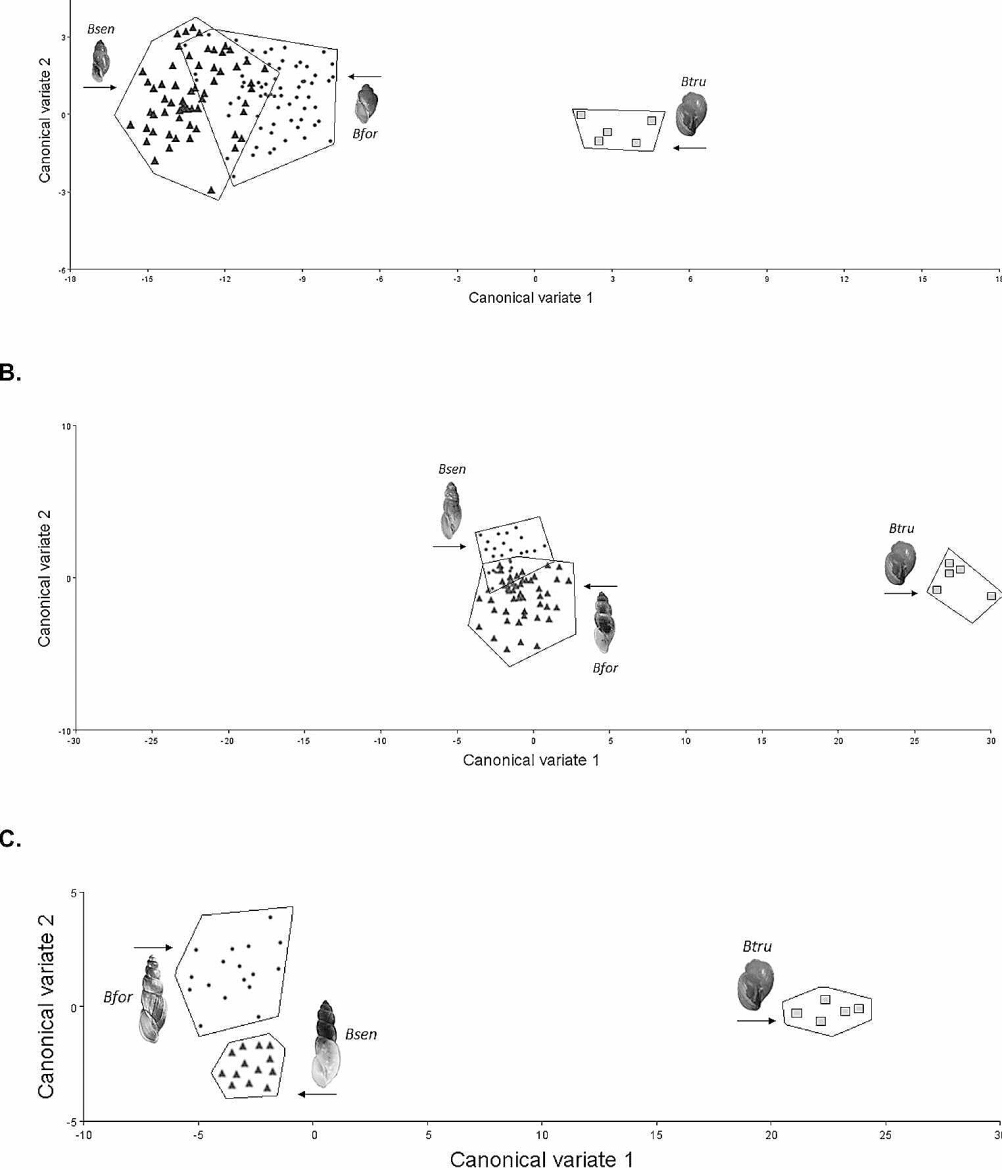
(**A**) CVA plot of 3-whorl *B. senegalensis* (*n* = 60) and *B. forskalii* (*n* = 66). CV1 explains 64.78% and CV2 explains 18.89% of variation. (**B**) plot of 4-whorl *B. senegalensis* (*n* = 48) and *B. forskalii* (*n* = 27). CV1 explains 81.04% and CV2 explains 13.31% of variation. (**C**) plot of 5-whorl *B. senegalensis* (*n* = 13) and *B. forskalii* (*n* = 17). CV1 explains 78.43% and CV2 explains 21.57% of variation. Adult *Bulinus truncatus* (*n* = 5) were also included in each analysis as a comparative outgroup

Canonical Variate Analysis (CVA) for 3-whorl shells grouped by species (identified using genetic methods and genital morphology), showed separation of *B. senegalensis and B. forskalii* with minimal overlap (CV1: 64.8% and CV2: 18.9%; Fig. 5a). Similarly, the CVA plot for 4-whorl shells grouped by species (identified using genetic methods and genital morphology), showed separation of *B. senegalensis and B. forskalii* again with minimal overlap (CV1: 81% and CV2: 13.3%; Fig. 5b). Finally, the CVA plot for 5-whorl shells grouped by species (identified using genetic methods and genital morphology), showed separation of *B. senegalensis and B. forskalii* with no overlap (CV1: 78.4% and CV2: 21.6%; Fig. 5c). Furthermore, the shape changes can be seen with the Canonical Variate transformation grids (Supplementary Fig. 1).

## Discussion

Shell morphology is a useful parameter for the identification of snail species due to its simplicity and low cost. However, if morphological identification is to be used in instances where morphologically similar looking species co-inhabit the same environment (as in the case of *B. senegalensis* and *B. forskalii*), more accurate techniques are required [30, 31]. Morphometrics has been previously used to help distinguish other medically important invertebrates such as Calliphoridae (Myiasis) [37], Culicidae (Malaria, Dengue, Chikungunya & Lymphatic filariasis) [38], Triatominae bugs (Trypanosomiasis) [39], and even other medically important gastropods, *Biomphalaria* (intestinal schistosomiasis) [28]. *Bulinus* shell morphology has been previously investigated using shell measurements and morphometrics [14, 15, 16–18, 27]. However, this is the first study that investigates the morphological differences between species, specifically the *B. forskalii* group. In this study, we used landmark based morphometrics, to distinguish two sister species of *Bulinus* (*B. senegalensis and B. forskalii*) at different developmental stages.

Canonical variate analysis showed varying levels of separation. 3-whorl juvenile shells showed some separation with minimal overlap. This separation becomes clearer in 4-whorl and 5-whorl shells which show complete separation between adult *B. senegalensis* and *B. forskalii*. This emphasizes the importance of stage of development on accuracy when trying to distinguish between *B. senegalensis* from *B. forskalii*.

**Fig. 6:**
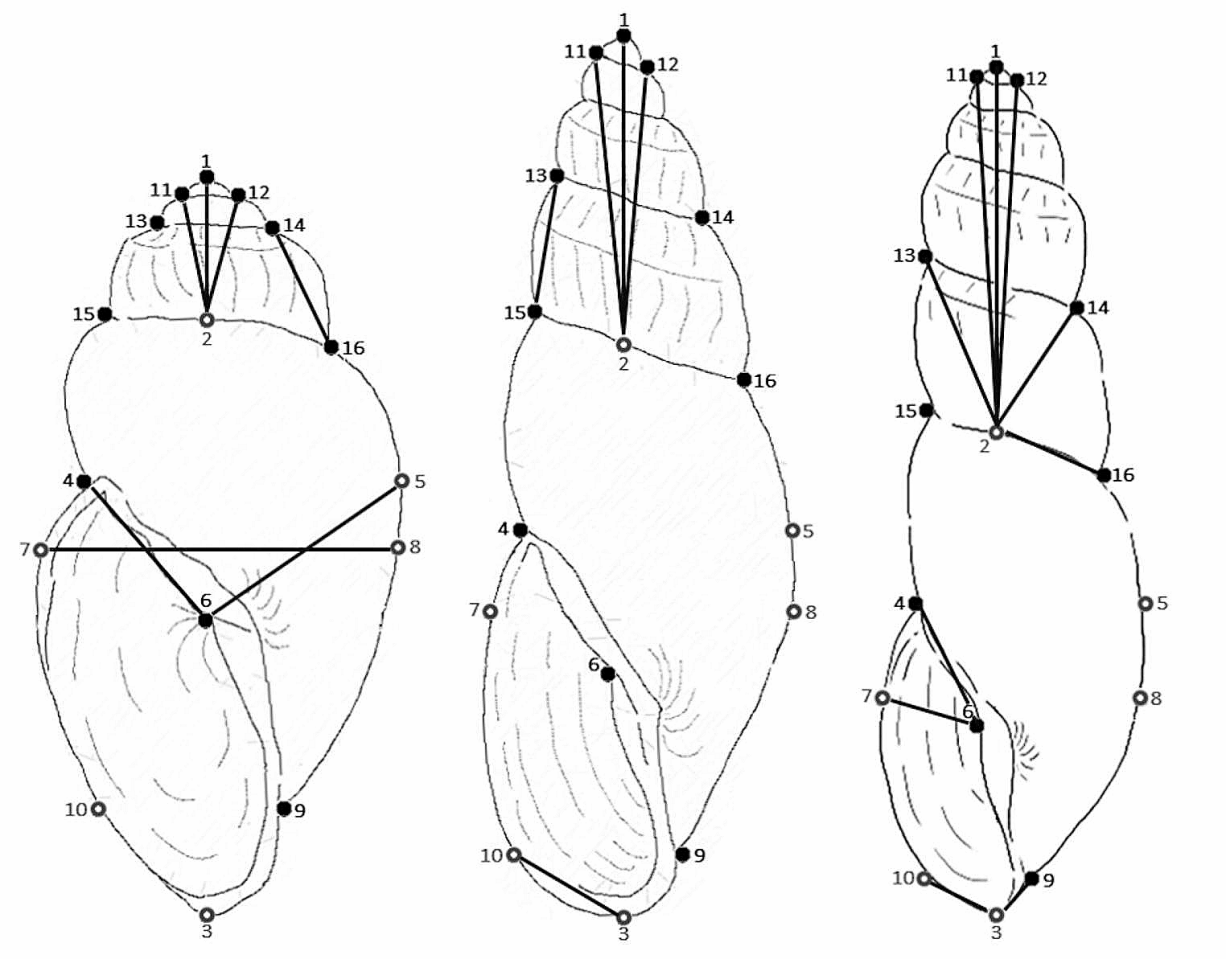
Significant landmark distances used to differentiate 3-whorl (left), 4-whorl (middle) and 5-whorl (right) *Bulinus senegalensis* shells from *B. forskalii* shells (not to scale)

Juvenile 3-whorl *Bulinus senegalensis* and *B. forskalii* were more similar in morphology than 4-whorl and 5-whorl shells. However, when the shells were scaled down to the same length (2 mm), the distance between landmarks showed clear differences. Juvenile 3-whorl *B. senegalensis* had a longer distance from L1 to L2, L2 to L11 and L2 to L12 of the spire; a longer distance from L14 to L16 of the penultimate whorl; a narrower distance from L5 to L6 and L6 to L8 of the body whorl; a shorter distance from L4 to L6 and L6 to L7 of the aperture than *B. forskalii*. Through the use of photomicrography, these significant measurements can be used to distinguish 3-whorl *B. senegalensis* from *B. forskalii* (Fig. 6).

The next development stage, 4-whorl shells, had fewer significant measurements than 3-whorl shells. When scaled down to the same length (4 mm), 4-whorl *B. senegalensis* had a longer distance from L1 to L2, L2 to L11 and L2 to L12 of the spire; a longer distance from L13 to L15 of the penultimate whorl and a shorter distance from L3 to L10 of the aperture than *B. forskalii*. The observation of fewer significant measurements for 4-whorl shells compared to the 3-whorl shells indicates that as *B. senegalensis* and *B. forskalii* progress from 3-whorl to 4-whorl, the shells become more similar. Developmental stage can affect how similar *B. senegalensis* and *B. forskalii* look [29]. However, spire length and aperture size can still be used to distinguish *B. senegalensis* from *B. forskalii* at this stage of development.

Adult 5-whorl shells had the most significant measurements of all stages. However, some of the trends of the previous two stages were the opposite. When scaled down to the same length (6 mm), 5-whorl *B. senegalensis* had a shorter distance from L1 to L2, L2 to L11 and L2 to L12 of the spire; a shorter distance from L2 to L13 and L2 to L14 of the penultimate whorl than *B. forskalii*. However, *B. senegalensis* still had a shorter distance from L2 to L16 of the body whorl and a shorter distance from L3 to L9, L3 to L10, L4 to L6 and L6 to L7 of the aperture than *B. forskalii*.

To summarise, *B. senegalensis* consistently had a significantly narrower body whorl and shorter aperture size than *B. forskalii*, regardless of whorl number. Spire length and penultimate whorl length can also be used to differentiate *B. senegalensis* and *B*. *forskalii*. However, our morphological technique has several limitations such as the need of a microscope due to the small size of the shells; the need to identify the developmental stage of the snail before identifying it; and our sampling only covers a small number of populations from specific African countries. Overall, landmark based morphometrics is helpful in improving the effectiveness and viability of using shell morphology as a method to differentiate *B. senegalensis* and *B. forskalii*. The following measurements L1–L2, L2–L11 and L2–L12 of the spire; L2–L13, L2–L14, L13–L15 and L14–L16 of the penultimate whorl; L2–L16, L5–L6 and L6–L8 of the body whorl; L3–L9, L3–L10, L4–L6 and L6–L7 of the aperture are useful to differentiate to differentiate *B. senegalensis* and *B. forskalii* (see Fig. 6). These measurements are sufficient to distinguish *B. senegalensis* and *B. forskalii* when used with the appropriate information for each development stage. More information about key landmark measurements expressed as ratios using shell length (L1-L3) for *B. senegalensis* and *B. forskalii* shells can be found in supplementary Table 3.

## Conclusion

We show that landmark based, morphometric analysis provides an effective and viable method for the differentiation of *Bulinus senegalensis* and *B. forskalii*. The accuracy of identifying a *Bulinus* species is affected by the stage of development (3-whorl, 4-whorl and 5-whorl). When scaled down to the same length, *B. senegalensis* shells consistently had a significantly shorter aperture size and body whorl width than *B. forskalii*. While spire length and penultimate whorl length were dependent on the development stage, with 3-whorl and 4-whorl *B. senegalensis* having a longer spire/penultimate whorl than *B. forskalii*. Conversely, adult 5-whorl *B. senegalensis* had a shorter spire/penultimate whorl than *B. forskalii*. We recommend using spire length, penultimate whorl length, and body whorl width (when used with the appropriate information for each development stage) in conjunction with aperture size (can be used at any development stage) to differentiate *B. senegalensis* and *B. forskalii*.

### Electronic Supplementary Material

Below is the link to the electronic supplementary material.

Supplementary Material 1

## Data Availability

All Photographical data is available upon request.
