# Supplementary material for: Differentiation of Bulinus senegalensis and Bulinus forskalii Snails in West Africa Using Morphometric Analysis
Source: Acta Parasitol. 2024 Mar 19;69(1):1016–26. doi: 10.1007/s11686-024-00830-1 (PMC11001693; doi:10.1007/s11686-024-00830-1)
Supplement: Supplementary file 1 — Supplementary Material 1 [file 11686_2024_830_MOESM1_ESM.docx]

# Supplementary:

**
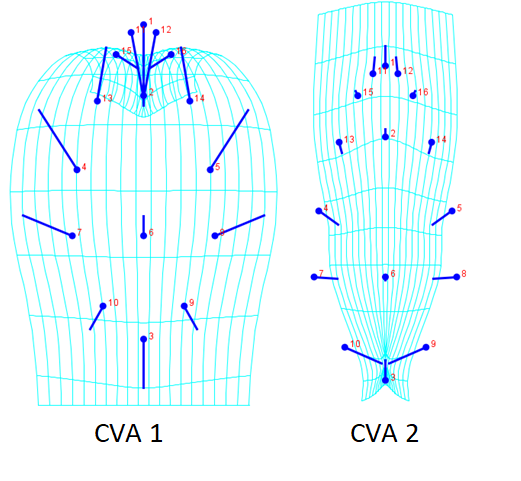
**

**A.**

**
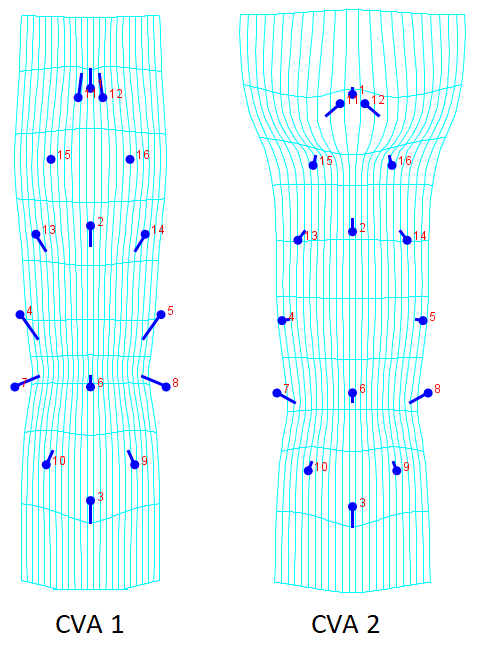
**

**B.**

**
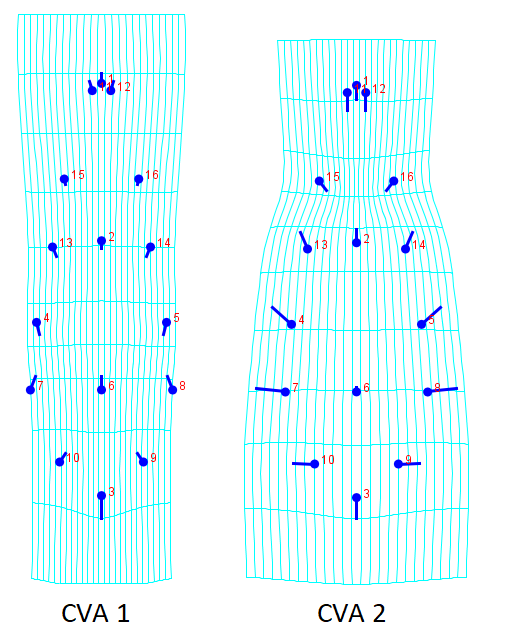
**

**C.**

Supplementary figure 1. Transformation grid of CV shape changes of (A) 3-whorl juvenile, (B) 4-whorl and (C) 5-whorl adult shells. The lines indicate the changes in the relative position of the landmarks for each Canonical Variate Axis (CVA).

Supplementary table 1. Mean lengths (L1-L3) and widths (L7-L8) (+ Standard deviation) of *Bulinus* collected from The Gambia.

|  | **Species** | **Location** | **Mean Length** **(mm) (+ SD)** | | | **Mean Width (mm) (+ SD)** | | |
| --- | --- | --- | --- | --- | --- | --- | --- | --- |
|  |  |  | **No. of Whorls** | | | **No. of Whorls** | | |
|  |  |  | **3** | **4** | **5** | **3** | **4** | **5** |
| **1** | *B. senegalensis* | Bajakunda | 4.81 (0.23) | 9.22 (0.34) | - | 2.07 (0.08) | 3.61 (0.27) | - |
| **2** | *B. senegalensis* | Changai 2 | - | 9.33 (0.32) | 10.46 (0.67) | - | 3.24 (0.19) | 3.45 (0.2) |
| **3** | *B. senegalensis* | Diabugu Basilla | 6.34 (0.28) | 6.7 (0.47) | - | 2.68 (0.12) | 2.69 (0.33) | - |
| **4** | *B. senegalensis* | Kuwonku | 5.58 (0.42) | 7.07 (0.74) | - | 2.03 (0.22) | 2.57 (0.31) | - |
| **5** | *B. senegalensis* | Madina Nfally 2 | 5.62 (0.27) | 6.31 (0.44) | - | 2.43 (0.13) | 2.46 (0.17) | - |
| **6** | *B. senegalensis* | Sare Jabel | 5.39 (0.79) | - | - | 2.29 (0.3) | - | - |
| **7** | *B. senegalensis* | Sare Madi Ganteh | 5.61 (0.33) | 7.1 (0.51) | - | 2.19 (0.16) | 2.44 (0.18) | - |
| **8** | *B. senegalensis* | Sutukonding | - | 6.94 (0.48) | 9.1 (0.34) | - | 2.41 (0.17) | 2.74 (0.16) |
| **1** | *B. forskalii* | Bansang | 3.11 (0.40) | - | - | 1.66 (0.10) | - | - |
| **2** | *B. forskalii* | Basse Kabakama | 3.53 (0.60) | - | - | 1.93 (0.15) | - | - |
| **3** | *B. forskalii* | Choya | 2.83 (0.40) | 4.81 (0.39) | - | 1.55 (0.19) | 1.65 (0.09) | - |
| **4** | *B. forskalii* | Dalaba | 2.34 (0.60) | - | - | 1.47 (0.22) | - | - |
| **5** | *B. forskalii* | Kuntaur | 3.89 (0.47) | - | - | 1.76 (0.14) | - | - |
| **6** | *B. forskalii* | Misra Ba Mariama | 3.37 (0.36) | - | - | 1.58 (0.11) | - | - |
| **7** | *B. forskalii* | Sotuma Sire | 2.18 (0.23) | 5.71 (0.15) | - | 1.22 (0.07) | 1.81 (0.08) | - |
| **1** | *B. truncatus* | Pacharr | 8.29 (0.92) | - | - | 5.93 (0.6) | - | - |

Supplementary table 2. Mean lengths (L1-L3) and widths (L7-L8) (+ Standard deviation) of Bulinus from the London Natural History Museum collections.

|  | **Species** | **Country** | **Mean Length (mm) (+ SD)** | | | **Mean Width (mm) (+ SD)** | | |
| --- | --- | --- | --- | --- | --- | --- | --- | --- |
|  |  |  | **No. of Whorls** | | | **No. of Whorls** | | |
|  |  |  | **3** | **4** | **5** | **3** | **4** | **5** |
| **1** | *B. senegalensis* | The Gambia | 4.3 (0.83) | 5.44 (0.21) | 10.21 | 2.04 (0.32) | 2.06 (0.18) | 3.34 |
| **2** | *B. senegalensis* | Nigeria | 2.79 (0.25) | - | 8.01 | 1.25 (0.22) | - | 2.51 |
| **3** | *B. senegalensis* | Senegal | - | 8.54 (0.53) | 9.79 | - | 3.19 (0.26) | 3.32 |
| **1** | *B. forskalii* | Angola | - | 6.17 (0.51) | 9.23 (1.28) | - | 2.84 (0.4) | 2.98 (0.48) |
| **2** | *B. forskalii* | Democratic Republic  of Congo | - | - | 10.79 (0.33) | - | - | 3.37 (0.19) |
| **3** | *B. forskalii* | Egypt | - | - | 9.99 (1.38) | - | - | 3.46 (0.23) |
| **4** | *B. forskalii* | Ethiopia | 7.63 (1.21) | 8.52 (0.36) | - | 3.02 (0.51) | 3.26 (0.05) | - |
| **5** | *B. forskalii* | The Gambia | 5.74 (0.67) | 6.24 (0.05) | - | 2.71 (0.42) | 2.21 (0.09) | - |
| **6** | *B. forskalii* | Kenya | 6.05 (0.85) | - | - | 2.28 (0.13) | - | - |
| **7** | *B. forskalii* | Mauritius | 5.03 (0.07) | - | - | 2.83 (0.13) | - | - |
| **8** | *B. forskalii* | Rwanda | 6.45 | - | - | 2.6 | - | - |
| **9** | *B. forskalii* | S. Africa | - | 5.23 (0.12) | - | - | 1.88 (0.14) | - |
| **10** | *B. forskalii* | S. Sudan | 6.4 | - | - | 2.75 | - | - |
| **11** | *B. forskalii* | Senegal | 7.05 | 7.55 (0.03) | 9.91 (1.01) | 2.75 | 3.32 (0.22) | 3.69 (0.35) |
| **12** | *B. forskalii* | Seychelles | 8.35 | - | 9.81 (0.8) | 3.5 | - | 3.7 (0.58) |
| **13** | *B. forskalii* | Sierra Leone | 6.08 (0.18) | - | - | 2.5 (0.05) | - | - |
| **14** | *B. forskalii* | Somalia | 4.85 | 6.8 (0.1) | - | 2.1 | 2.48 (0.14) | - |
| **15** | *B. forskalii* | Tanzania | - | - | 9.89 (0.54) | - | - | 3.06 (0.28) |
| **16** | *B. forskalii* | Uganda | - | 6.91 (0.43) | 10.43 | - | 2.74 (0.2) | 3.24 |

Supplementary table 3. Mean distances measured between significant landmarks (+ Standard deviation) of 3-whorl, 4-whorl and 5-whorl *B. senegalensis* and *B. forskalii* shells scaled down to the same length (3W=2mm; 4W= 4mm; 5W=6mm). Ratios of mean landmark distances to mean shell length (L1-L3) for *B. senegalensis* and *B. forskalii* shells.

| 3-whorl | | | | | | | |
| --- | --- | --- | --- | --- | --- | --- | --- |
| *B. senegalensis* | | | | *B. forskalii* | | | |
| LM | Mean | SD | Length Ratio | LM | Mean | SD | Length Ratio |
| 1-2 | 0.53 | 0.05 | 3.8 : 1 | 1-2 | 0.40 | 0.06 | 5 : 1 |
| 2-11 | 0.48 | 0.05 | 4.2 :1 | 2-11 | 0.37 | 0.06 | 5.4 : 1 |
| 2-12 | 0.47 | 0.05 | 4.3 : 1 | 2-12 | 0.36 | 0.06 | 5.5 : 1 |
| 4-6 | 0.47 | 0.02 | 4.3 : 1 | 4-6 | 0.53 | 0.05 | 3.8 : 1 |
| 5-6 | 0.59 | 0.04 | 3.4 : 1 | 5-6 | 0.68 | 0.05 | 2.9 : 1 |
| 7-8 | 0.43 | 0.05 | 4.6 : 1 | 6-8 | 0.50 | 0.04 | 4 : 1 |
| 14-16 | 0.42 | 0.02 | 4.8 : 1 | 14-16 | 0.35 | 0.04 | 5.7 : 1 |

| 4-whorl | | | | | | | |
| --- | --- | --- | --- | --- | --- | --- | --- |
| *B. senegalensis* | | | | *B. forskalii* | | | |
| LM | Mean | SD | Length Ratio | LM | Mean | SD | Length Ratio |
| 1-2 | 1.49 | 0.10 | 2.7 : 1 | 1-2 | 1.28 | 0.06 | 3.1 : 1 |
| 2-11 | 1.42 | 0.10 | 2.8 : 1 | 2-11 | 1.23 | 0.05 | 3.2 : 1 |
| 2-12 | 1.41 | 0.10 | 2.8 : 1 | 2-12 | 1.19 | 0.04 | 3.4 : 1 |
| 3-10 | 0.58 | 0.10 | 6.9 : 1 | 3-10 | 0.80 | 0.05 | 5 : 1 |
| 13-15 | 0.75 | 0.06 | 5.3 : 1 | 13-15 | 0.65 | 0.03 | 6.1 : 1 |

| 5-whorl | | | | | | | |
| --- | --- | --- | --- | --- | --- | --- | --- |
| *B. senegalensis* | | | | *B. forskalii* | | | |
| LM | Mean | SD | Length Ratio | LM | Mean | SD | Length Ratio |
| 1-2 | 2.25 | 0.10 | 2.7 : 1 | 1-2 | 2.68 | 0.10 | 2.2 : 1 |
| 2-11 | 2.15 | 0.12 | 2.8 : 1 | 2-11 | 2.58 | 0.10 | 2.3 : 1 |
| 2-12 | 2.14 | 0.11 | 2.8 : 1 | 2-12 | 2.52 | 0.10 | 2.4 : 1 |
| 2-13 | 0.55 | 0.03 | 11 : 1 | 2-13 | 0.58 | 0.03 | 10 : 1 |
| 2-14 | 0.86 | 0.05 | 7 : 1 | 2-14 | 0.89 | 0.09 | 6.7 : 1 |
| 2-16 | 1.05 | 0.04 | 5.7 : 1 | 2-16 | 1.02 | 0.04 | 5.9 : 1 |
| 3-9 | 0.73 | 0.04 | 8.2 : 1 | 3-9 | 0.77 | 0.06 | 7.8 : 1 |
| 3-10 | 0.89 | 0.09 | 6.7 : 1 | 3-10 | 0.92 | 0.09 | 6.5 :1 |
| 4-6 | 1.15 | 0.04 | 5.2 : 1 | 4-6 | 1.22 | 0.08 | 4.9 : 1 |
| 6-7 | 0.85 | 0.03 | 7 : 1 | 6-7 | 0.91 | 0.07 | 6.6 : 1 |
